# The GET READY relapse prevention programme for anxiety and depression: a mixed-methods study protocol

**DOI:** 10.1186/s12888-019-2034-6

**Published:** 2019-02-11

**Authors:** Esther Krijnen-de Bruin, Anna D. T. Muntingh, Adriaan W. Hoogendoorn, Annemieke van Straten, Neeltje M. Batelaan, Otto R. Maarsingh, Anton J. L. M. van Balkom, Berno van Meijel

**Affiliations:** 1 Department of Health, Sports & Welfare, Cluster Nursing, Inholland University of Applied Sciences, Research Group Mental Health Nursing, Amsterdam, The Netherlands; 2 Amsterdam UMC, Vrije Universiteit, Psychiatry, Amsterdam Public Health research institute, Amsterdam, The Netherlands; 3 GGZ inGeest Specialized Mental Health Care, Amsterdam, The Netherlands; 4 Department of Clinical Psychology, Faculty of Behavioural and Movement Sciences, Amsterdam UMC, Vrije Universiteit Amsterdam, Amsterdam Public Health research institute, Van der Boechorststraat 1, 1081 BT Amsterdam, the Netherlands; 5 Department of General Practice & Elderly Care Medicine, Amsterdam UMC, Vrije Universiteit Amsterdam, Amsterdam Public Health research institute, De Boelelaan, 1117 Amsterdam, The Netherlands; 6 Parnassia Psychiatric Institute, Parnassia Academy, The Hague, The Netherlands; 7 GGZ-VS Academy for Masters in Advanced Nursing Practice, Utrecht, The Netherlands

**Keywords:** Anxiety, Depression, Recurrence, Relapse prevention, Self-management, E-health, Mixed-methods research, Study protocol

## Abstract

**Background:**

Since anxiety and depressive disorders often recur, self-management competencies are crucial for improving the long-term course of anxiety and depressive disorders. However, few relapse prevention programmes are available that focus on improving self-management. E-health combined with personal contact with a mental health professional in general practice might be a promising approach for relapse prevention. In this protocol, the GET READY (Guided E-healTh for RElapse prevention in Anxiety and Depression) study will be described in which a relapse prevention programme is developed, implemented and evaluated. The aim of the study is to determine patients’ usage of the programme and the associated course of their symptoms, to examine barriers and facilitators of implementation, and to assess patients’ satisfaction with the programme.

**Methods:**

Participants are discharged from mental healthcare services, and are in complete or partial remission. They receive access to an E-health platform, combined with regular contact with a mental health professional in general practices. Online questionnaires will be completed at baseline and after 3, 6 and 9 months. Also, semi-structured qualitative individual interviews and focus group interviews will be conducted with patients and mental health professionals.

**Discussion:**

This mixed-methods observational cohort study will provide insights into the use of a relapse prevention programme in relation to the occurrence of symptoms, as well as in its implementation and evaluation. Using the results of this study, the relapse prevention programme can be adapted in accordance with the needs of patients and mental health professionals. If this programme is shown to be acceptable, a randomized controlled trial may be conducted to test its efficacy.

**Trial registration:**

Retrospectively registered in the Netherlands Trial Register (NTR7574; 25 October 2018).

**Electronic supplementary material:**

The online version of this article (10.1186/s12888-019-2034-6) contains supplementary material, which is available to authorized users.

## Background

The course of anxiety and depressive disorders is often unfavourable, with chronic [1, 2] or intermittent episodes of anxiety and/or depression [3] and a high risk of relapse. Percentages of 22–58 for anxiety [1, 3–5] and 27–77 for depression [6–9] have been reported as relapse rates with higher rates in longer follow-up studies [1, 6]. Furthermore, patients often achieve partial remission instead of full remission, and have residual symptoms [10]. The risk of relapse is higher for patients with a partial remission when compared to patients with full remission [11]. Moreover, even if remission is achieved, patients with residual symptoms have a three times higher risk of relapse than patients without residual symptoms [12]. Since anxiety and depression are often recurrent and patients remain vulnerable, these disorders should be approached as chronic diseases. Chronic care models, previously developed for other chronic diseases such as diabetes [13], can be applied. One essential element of chronic care models is the support of self-management [14]. Lorig [15] defined self-management as “learning and practicing skills necessary to carry on an active and emotionally satisfying life in the face of a chronic condition” (p.11). Self-management for anxiety and depression focuses on recovery and stabilisation of symptoms, prevention of relapse, and improving functioning and quality of life [16, 17]. People with recurrent anxiety and depression should be supported in performing self-management strategies.

One of the major challenges in the management of anxiety and depression symptoms is the prevention of relapse [18]. In their meta-analysis, Biesheuvel-Leliefeld et al. [19] concluded that relapse prevention strategies such as cognitive therapy or mindfulness-based cognitive therapy are effective in reducing relapse of depression. However, few relapse prevention programmes are available for this patient group of depressed patients. In an empirical study from the same authors, the efficacy of a self-help preventive cognitive therapy combined with weekly telephone guidance in preventing depression was examined [20]. They demonstrated that this was significantly more effective than care as usual. To date, little is known about the efficacy of relapse prevention strategies for patients with anxiety disorders [21]. The fact that depression and anxiety are often comorbid, and relapse into another disorder frequently occurs (from anxiety to depression and vice versa), highlights the need to target both anxiety and depression in a single relapse prevention programme.

Over the past two decades, it has been suggested that primary healthcare facilities should play a vital role in the long-term treatment of patients with anxiety and depression [22, 23]. In the Netherlands, primary mental healthcare is usually provided by a mental health professional (MHP), working in a general practice and reporting to the general practitioner (GP). This MHP could be a (community) mental health nurse, a social worker or a (junior) psychologist. Since general practices are always located close to where patients live, and the services are freely available, the MHPs are easily approachable. They can play a pivotal role in the monitoring of symptoms and the support of self-management, and therefore in the prevention of relapse. However, many MHPs are unfamiliar with relapse prevention interventions, including the use of supporting tools to monitor symptoms [24]. To encourage general practices to offer structured relapse prevention, while also supplying supporting tools for MHPs, the use of E-health could offer a solution. E-health tools can be easily tailored to the needs and preferences of patients, which is important for the tools’ acceptability and effectiveness. Most (92%) of the MHPs in the Netherlands are already familiar with E-health [25]. If the use of E-health is embedded in personal contact with a MHP, it is likely to be more effective [26].

We therefore developed a relapse prevention programme that can be offered in general practices by MHPs to patients with (partially) remitted anxiety or depression. This programme aims to support self-management skills and provides tools for monitoring symptoms. The E-health modules can be individually tailored to the needs of patients, and is combined with regular contact with the MHP. The aim of the present study is to implement and evaluate this guided self-help online relapse prevention programme for patients who are completely or partially in remission from anxiety and/or depressive disorders, and who previously received treatment in mental healthcare services. This study will provide insight into: 1) the extent to which patients make use of the relapse prevention programme; 2) the factors that influence the use of the programme; 3) the association between usage intensity and course of symptoms; 4) barriers and facilitators in implementation of the programme; and 5) how patients evaluate the programme.

## Methods/design

The methods section is divided into three parts: 1) the development of the relapse prevention programme; 2) the content of the relapse prevention programme; and 3) the study design.

### Development of the relapse prevention programme

The programme was developed using input from different studies. Muntingh et al. [27] examined preferences of patients regarding relapse prevention and revealed that patients prefer a relapse prevention programme that is effective but not too time-consuming, taking up a maximum of 1 h a week. Using a personal relapse prevention plan, in combination with regular contact with a professional (about once every 3 months) was the most preferred relapse prevention strategy. The use of a relapse prevention plan as a practical aid for the early recognition and management of potential triggers and signs that indicate relapse is in accordance with the NICE guidelines [28]. According to several studies, relapse prevention should be flexible and tailored to the patient’s individual situation and preferences in order to increase acceptability [29, 30]. Our online programme therefore consists of a personal relapse prevention plan and flexible E-health modules aiming at the promotion of self-management skills. The MHP can individually tailor the programme in conjunction with the patient.

The initial format and underpinning of the relapse prevention programme was discussed with academic experts, a panel of eight patients, and four MHPs. The content of the E-health modules was written by two expert psychologists, based on the principles of cognitive behavioural therapy (CBT). This therapy is effective in the treatment of anxiety and depression [31], and in preventing relapse in depression [19]. Some optional modules were added at the request of patients, such as healthy food and physical exercise. These modules contain psychoeducation and the ability to plan healthy behaviour, with the aim of increasing physical and mental health. Preliminary versions of the E-health modules were reviewed by the members of the research team and E-health developers. Also, the patient panel reviewed the modules and provided feedback. This feedback was thoroughly discussed and processed by the researchers. For example: more lengthy text parts were placed in ‘read more’ menus, an overview page was created, and the possibility to print text was added. Finally, the adjusted content was released to the online platform.

### Content of relapse prevention programme

#### Online programme

**Fig. 1:**
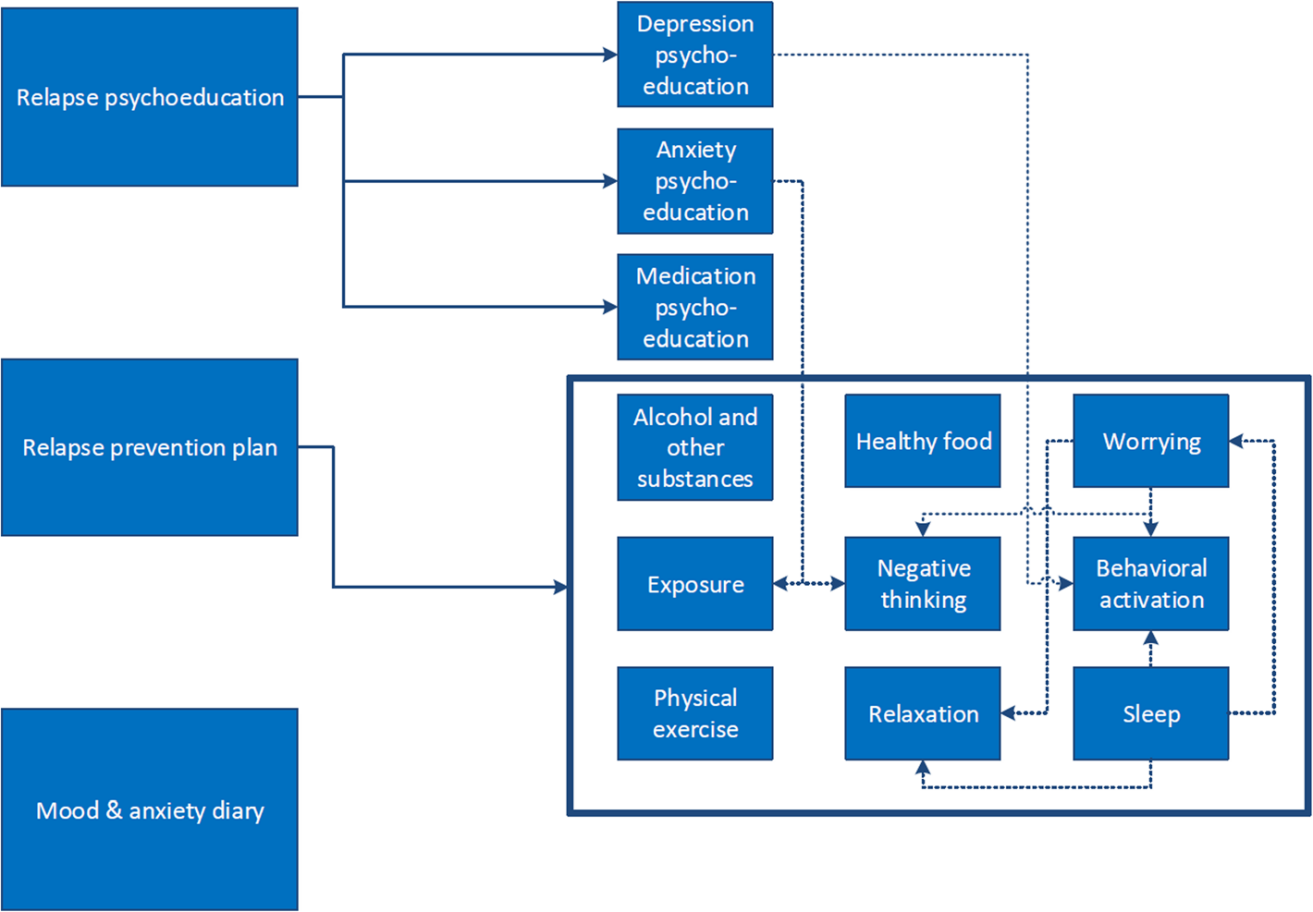
Content of E-health programme

The online programme consists of three basic components and 12 optional modules (see Fig. 1). The three basic components are: ‘Relapse psychoeducation’, ‘Relapse prevention plan’, and ‘Mood & anxiety diary’. As soon as the patient completes the module ‘Relapse psychoeducation’, the optional follow-up psychoeducation modules ‘Depression’, ‘Anxiety’ and ‘Medication’ will appear. Following the module ‘Relapse prevention plan’, the patient can choose which other optional modules he or she wishes to complete, based on own preferences and goals, thus offering the opportunity for the patient to customise treatment. The dotted lines in Fig. 1 indicate that certain modules refer automatically to other optional follow-up modules.

The modules include short videos on what to expect in the module, written information, exercises, and clinical examples of fictional patients. In all modules except the psychoeducation modules, patients have the possibility to ask for feedback from the MHP. For a more detailed overview of the contents of the E-health programme, see Table 1.

**Table 1:** Overview of module content

Module	Module content
Relapse	Information regarding relapse and relapse prevention Video: experiences of patients Overview of relapse prevention programme
Relapse prevention plan	Formulating a relapse prevention plan Information about support from family/friends Choosing which optional modules to complete
Depression	Information regarding symptoms, differences in disorders, causes and prevalence of depression Links to websites for more information
Anxiety	Information regarding symptoms, differences in disorders, causes and prevalence of anxiety Links to websites for more information
Medication	Information regarding antidepressive medication Information regarding benzodiazepines
Alcohol and other substances	Information regarding use of alcohol and psychological symptoms Advice on how to reduce alcohol intake Information regarding other substances (marihuana and tobacco) and psychological symptoms Exercise: registering use of substances Exercise: motivation to change behaviour Plan to change behaviour
Exposure	Information on exposure Exercise: avoidance Plan for exposure
Physical exercise	Information regarding exercise Plan to increase physical exercise Links to websites for more information
Healthy food	Information on healthy food Advice on healthy food Plan to improve your diet
Negative thinking	Information on (un)helpful thoughts Exercise: make a thought schedule
Relaxation	Information regarding physical and psychological effects of relaxation Information regarding mindfulness Exercises in mindfulness Plan to increase mindfulness/relaxation
Worrying	Exercise and information on registering worrying Exercises on worrying
Behavioural activation	Information on importance of behavioural activation Information regarding physical and psychological effects of pleasurable activities Exercise: planning pleasurable activities
Sleep	Information regarding sleep and psychological symptoms Advice on how to improve sleep Exercise: completing a sleep diary Exercise: restoring your sleep rhythm Exercise: activities during the day

It takes about 30–60 min to complete each module. In each module, patients draft plans related to the specific content of that module, and are encouraged to continue practising, by offering the possibility to print out the plans.

Patients receive reminders via email regarding the completion of modules in the E-health platform. They also receive a newsletter every 6 weeks to keep them involved in the programme by providing information about numbers of included patients, experiences of other patients and interesting articles or facts about anxiety and depression. The MHP has access to the patient’s account, and can check whether the patient has been using the diary to monitor symptoms of anxiety and depression. Also, the MHP can monitor the patient’s individual use of the different modules. In turn, MHPs can provide feedback on the completed modules and start a conversation in the E-health platform with the patient.

#### Role of MHP

The MHP and the patient will have at least one face-to-face contact during the nine-month period of the study, and are encouraged to meet each other every 3 months. During the first contact, the patient and MHP will start drawing up the relapse prevention plan (if not yet available) and decide on the frequency and number of contacts. In the follow-up contacts, the outcomes of the ‘mood and anxiety diary’, the use of the available modules and possible questions will be discussed. The time required for the first contact is 45 min, and 20 min for the follow-up contacts. The MHP can actively support the patient in using the online programme and provide online feedback at the request of the patient.

### Study design

In this mixed-methods observational cohort study, the relapse prevention programme we developed will be implemented and evaluated. This programme is targeted at patients who have been discharged from mental healthcare services, and are in complete or partial remission from an anxiety or depressive disorder. This relapse prevention programme is called ‘GET READY’ and offers access to an E-health platform, combined with regular contact with a MHP for a period of 9 months. General practices will be included in this study. Eligible patients completed mental health treatment for anxiety or depression and are in (partial) remission. Patients will complete online questionnaires at baseline, after 3, 6 and 9 months. In addition, individual interviews and focus group interviews will be held with patients as well as MHPs to evaluate the programme and its implementation.

### Definition of terms

Throughout the literature, several terms are used regarding relapse and remission. Based on the definitions described by Frank et al. [32], we will make use of three terms: the term ‘relapse’ refers to a return of full symptomatology in concordance with the DSM-IV criteria for a depressive or anxiety disorder [33]. The term ‘full remission’ indicates that no more than minimal symptoms are present and DSM-IV criteria for a disorder have not been fulfilled. In addition, ‘partial remission’ also indicates that DSM-IV criteria for a disorder have not been fulfilled, but that more than minimal symptoms are present.

### Setting

This study will be performed in two settings:

1. General practices throughout the Netherlands, with the participation of approximately 50 MHPs. Patients follow the programme at home via an E-health platform, accompanied by face-to-face contact with the MHPs.
2. Ambulatory mental healthcare services with the participation of eligible patients whose GP is not willing to participate in this study. A trained MHP will deliver the relapse prevention programme using the same protocol as in the participating general practices.

### Recruitment of MHPs

We aim to recruit 50 MHPs throughout the Netherlands. Recruitment will be done via telephone, letters, advertisements on websites for MHPs, and via the professional networks of the researchers. For the MHPs working in a general practice, the GP agrees that the MHP participates in this study. Informed consent will be obtained from the MHPs before inclusion.

### Recruitment of participants

Patients will be recruited via general practices and mental healthcare services. Patients are eligible to participate when they have been treated for an anxiety disorder and/or a depressive disorder in mental healthcare services and are in full or partial remission, according to their clinician. To confirm the clinician’s judgement regarding remission status, the Inventory of Depressive Symptomatology (IDS-SR) and the Beck Anxiety Inventory (BAI) will be administered at baseline [34, 35]. Patients with scores > 39 on the IDS-SR and > 30 on the BAI are excluded from the study, since these scores indicate severe symptoms [36, 37]. Inclusion criteria are: patients have completed their treatment for anxiety and/or depression within the last 2 years, have a score on the Global Assessment of Functioning scale (GAF) of 50 or higher, are at least 18 years old, and have sufficient command of the Dutch language. Patients are excluded if they participate in another structured psychological intervention, when they do not have access to the internet, or when the severity of a comorbid psychiatric disorder requires specialised treatment.

#### Recruitment via general practices

Each MHP is requested to include all patients that completed mental health treatment for anxiety and depression and meet the inclusion criteria. MHPs will be asked to identify eligible patients through their patient files. MHPs invite potentially eligible patients for a consultation to discuss participation in the study, to provide information about the study and to check whether patients meet the inclusion criteria. If interest is shown in the study, the researcher contacts the patient and sends the baseline questionnaire. When patients have completed the baseline questionnaire, they receive access to the E-health platform. Eight days after sending the invitation for login, the researchers check if the patient has logged in. If not, they contact the patient to offer technical or practical support.

#### Recruitment via mental healthcare services

Patients who completed their treatment and are recruited via mental healthcare services, will be contacted by the researchers directly. These patients will be supported during the GET READY programme by a MHP working in the ambulatory mental healthcare.

### Implementing the relapse prevention programme

#### Training of the MHP

Each MHP participates in a four-hour training course, focusing on the background and relevance of relapse prevention in anxiety and depressive disorders and potential effective intervention strategies regarding relapse prevention. Next, the content and use of the E-health platform is explained. Finally, information is provided about the study protocol. This training course will be given by either a psychologist or psychiatrist (both part of the research team), together with the first author of this paper.

#### Support for the MHP

After completing the training course, MHPs will receive an information package, including a protocol in which the following topics are described: recruitment of patients, the content of the face-to-face contact with patients, instructions on how to complete the case registration forms, and a guide on the E-health platform, containing screenshots. The package also includes invitation and information letters for patients.

The researchers offer monthly individual consultation via phone to the MHPs to support them in recruiting patients, discuss possible issues, and answer additional questions. Every month the MHPs receive a newsletter to update them on the study and motivate them to continue including patients.

### Quantitative data

#### Data collection – measurements

Patients are asked to complete four online questionnaires: at baseline (T0), after 3 months (T3), after 6 months (T6), and after 9 months (T9). It takes approximately 20–30 min to complete the questionnaires. Patients receive email invitations and if they do not complete the questionnaire, weekly reminders are sent.

Next, patients are requested to complete the ‘mood & anxiety diary’ every week to rate their level of anxiety and depression. They can complete the diary on their smartphone or on a computer.

After each face-to-face contact, the MHP completes a case registration form. This form contains information on the duration and content of the face-to-face contact, clinical status description, and whether additional appointments were made.

#### Data collection – outcome measures

##### Use of relapse prevention programme

Data will be collected regarding the use of the E-health platform data (number of logins, number of completed/uncompleted modules, and number of diary entries) and the frequency of contact with the MHPs, registered via the case registration forms.

##### Demographics and clinical variables

Sociodemographic characteristics and clinical variables of patients will be assessed at baseline, see Table 2. In addition, patients are asked to estimate their risk of relapse, and to indicate the expected effect of the programme.

**Table 2:** Information on data collection

Research question	Type	Outcome measures	T0	T3	T6	T9	After study
1) To what extent do patients use a tailored relapse prevention programme?	Quantitative	Use of the relapse prevention programme (log data)	x	x	x	x	
		Case registration form					x
2) What factors influence the use of the programme?	Quantitative	Demographic variables: - Gender - Age - Nationality - Ethnicity - Socioeconomic status - Marital status - Education - Employment status	x				
		Clinical variables: - Earlier treatment of depression and/or anxiety - Number of previous episodes - Age of onset of symptoms - Family history of depression and/or anxiety - Estimated risk of relapse - Estimated effect of the programme	x				
		Self-management strategies	x			x	
3) What is the association between usage intensity and course of symptoms?	Quantitative	Use of the relapse prevention programme (log data)	x	x	x	x	
		Case registration form					x
		BAI	x	x	x	x	
		ASI	x	x	x	x	
		IDS-SR	x	x	x	x	
		WHODAS 2.0	x	x	x	x	
		TiC-P	x	x	x	x	
		Weekly mood and anxiety diary	x	x	x	x	
4) What are barriers and facilitators in the implementation of the programme?	Qualitative	Individual interviews					x
		Focus group interview					x
5) How do patients evaluate the programme?	Qualitative	Satisfaction with treatment measure				x	
		Individual interviews					x
		Focus group interview					x

##### Self-management strategies

Self-management strategies will be measured using the ‘Self-management in depression and anxiety’ questionnaire. This questionnaire was developed during a study on self-management interventions for chronic anxiety and depression [38]. It consists of 45 items describing strategies that patients use to cope with anxiety and depression. Each item is rated on a 5-point Likert scale ranging from 1 (not at all) to 5 (a lot). The total score can be calculated by summing the individual scores.

##### Anxiety and depression scores

Anxiety severity will be measured by the Beck Anxiety Inventory (BAI), a 21-item tool, in which each item is rated on a 0 to 3 point scale, resulting in a total score between 0 to 63, with ≥30 indicating severe anxiety [34]. This tool is reported to have a good reliability [39] and validity [40].

The Anxiety Sensitivity Index (ASI) is a 16-item questionnaire for measuring anxiety sensitivity. Each item is rated on a 5-point Likert scale with scores from 0 (barely) to 4 (very much), with a total score ranging from 0 (no anxiety sensitivity) to 64 (severe anxiety sensitivity) [41]. The ASI is a reliable measure [42]. Anxiety sensitivity has been found to be a predictor for relapse in patients with remitted anxiety disorders [3].

The Inventory of Depressive Symptomatology – self-report (IDS-SR) is a 30-item questionnaire for measuring severity of depressive symptoms [35]. Each item is rated on a 0 to 3-point scale, and by summing 28 of the 30 items the total score ranges from 0 to 84, with ≥39 indicating severe depressive symptoms. The questionnaire has highly acceptable psychometric properties [43].

Anxiety and mood data are also collected in the weekly diary. Mood is measured by asking ‘how would you rate your mood today?, from 1 (very sad) to 10 (very happy)’. Anxiety is measured by asking ‘how would you rate the intensity of anxiety today?, from 1 (very relaxed) to 10 (very anxious/tense)’.

##### Functioning

General functioning and disability will be measured using the World Health Organization Disability Assessment Schedule 2.0 (WHODAS 2.0). This 36-item tool measures six domains: cognition, mobility, self-care, getting along, life activities and participation. Each item is rated on a 5-point Likert scale with scores from 0 (no difficulty) to 4 (extreme difficulty or cannot do). By using the ‘item-response theory’-based scoring, the scores will range from 0 (no disability) to 100 (full disability) [44]. The WHODAS 2.0 is reported to have strong psychometric properties [45].

##### Healthcare use

Healthcare and medication use will be measured using an adjusted version of the Trimbos/iMTA Questionnaire for Costs Associated with Psychiatric Illness (TiC-P). Only psychotropic medication will be registered. Health care use is measured as the number of contacts with different healthcare providers. The TiC-P is a feasible and reliable instrument for measuring healthcare use and costs [46].

##### Satisfaction with the relapse prevention programme

Satisfaction with the relapse prevention programme will be assessed using the ‘Satisfaction with treatment measure’. This 14-item measure provides insight into overall satisfaction, how helpful and useful patients valued the programme, perceived support and effect [47]. Also patients will be asked to rate each online module. The original scale was translated into Dutch by the authors, with permission of the author.

#### Quantitative data analysis

For each of the quantitative research questions, a separate analysis will be performed using log data from the E-health platform, data from the case registration forms that have been completed by the MHPs, and data from the four questionnaires:

1. To what extent do patients use a tailored relapse prevention programme?

The use of the tailored relapse prevention programme will be described, focusing on different aspects of its use, such as the number of login sessions, the amount of time spent online, the number of completed modules, the number of diary entries, the number of sessions and the number of online and face-to-face conversations with the MHP. Descriptive statistics will be used to provide insight into these aspects of the use of the relapse prevention programme.

2) What factors influence the use of the programme?

The use of the tailored relapse prevention programme may differ across patients, and may depend on age, education level and clinical situation. We will explore possible differences in usage between groups of patients systematically, considering sociodemographic factors, clinical symptoms and self-management strategies.

3) What is the association between usage intensity and course of symptoms?

Descriptive statistics will be used to provide insight into course of symptoms for ‘high-use’ participants and ‘low-use’ participants. In order to analyse the association between usage intensity and course of symptoms, multiple regression analyses will be performed. We will first analyse the change in anxiety severity from baseline to follow-up (9 months) with the following independent variables: intensity of E-health use, number of diary entries and number of contacts with the MHP. We will repeat these analyses for the depression outcomes. In addition, analyses will be performed using ‘deterioration: yes or no’ as outcome variable. Deterioration is defined as an increase of 1 standard deviation on the IDS-SR and/or on the BAI. The standard deviation will be calculated using data from the NESDA study [48], the study from Kok et al. [49], and the present study. This analysis will be performed using a time-lag model, in which the outcome will be assessed using determinants from an earlier measurement (deterioration at time_*t*_ predicted by usage intensity at time_*t-1*_), since we assume that usage intensity might influence whether deterioration occurs at a later point in time.

Because of the observational nature of the data, it will be difficult to draw conclusions about the effects of the relapse prevention programme. However, we will conduct additional explorative analyses to estimate the association between usage intensity and course of symptoms. All data analysis will be performed using SPSS statistical analysis software.

#### Sample size

In order to determine the sample size, we assumed a moderate effect (*r* = 0.24 that corresponds to Cohen’s *d* = 0.5) of the intensity of programme use (*X*) on the change in symptoms of anxiety and depression (*Y*). Since this is a non-randomized study, we will use a set of covariates ***W*** to correct for selectivity in the uptake of the programme. In applying the G*Power 3 software [50] to determine the sample size, we assume that the effect of *X* corresponds to a 6% of explained variance of *Y* (equivalent to the moderate effect, since *R*^2^ = *r*^2^ = 0.24^2^ = 0.06) above the covariates ***W*** and assume a 6% reduction of the total variance in *Y* of the residual variance due to the use of covariates of ***W***, leading to a partial *R*^2^ = 0.06 or, equivalently, an effect size of *f*^2^ = 0.0638. Furthermore, setting *α* = 0.05 and the power of 1 – *β* = 0.80, the sample size calculation shows that 126 patients are needed. Patients will be considered as drop-out when they refuse to complete questionnaires. With an estimated attrition of 20% (i.e. 80% complete at least one follow-up questionnaire), we aim to include 158 patients.

### Qualitative data

#### Data collection – measurements

The individual interviews and focus group interviews are conducted to assess implementation and satisfaction with the programme.

##### Individual interviews

Paired interviews will be conducted separately with patients and their MHPs in order to gain insight into both perspectives on one case. These paired interviews provide valuable insights regarding similarities and differences in experiences of MHPs and patients [51, 52]. Purposive sampling will be used to select patients and realise sufficient variation in our sample with respect to gender, age, severity of symptoms, and use of the programme [53]. Patients will be invited by email and telephone to participate in a semi-structured interview to evaluate their experience with the relapse prevention programme. If willing to participate, their MHP will be invited as well. The interviews will be conducted by two researchers, who will prepare the interviews by performing two test interviews. A senior researcher will provide supervision and participate in the analysis. We aim to conduct 12–15 interviews with patients and 12–15 interviews with MHPs. The final number of interviews depends on when data saturation is achieved.

A topic guide is developed to support the interview process [see Additional file 1]. This guide was drafted by the first author and reviewed by two experts, and inspired by the Consolidated Framework for Implementation Research (CFIR) [54]. Main topics are: experiences with the relapse prevention programme, use of the relapse prevention programme, useful and less useful aspects of the programme, and suggestions to improve the programme. Input from the E-health platform will be used, such as completed and uncompleted modules, use of the diary, and number of online conversations with the MHP. The topic guide will be evaluated and updated after conducting four interviews.

The MHPs of patients who participate in the interviews will also be invited by email and telephone to participate in an interview. Besides evaluating the programme (research question 5), implementation barriers and facilitators will be discussed in these interviews (research question 4), see Additional file 2 for the topic guide.

These interviews will be conducted in a setting selected by participants and will take approximately 45 min.

##### Focus group interviews

After completing the individual interviews, two focus group interviews will be conducted, one with 8–10 patients and one with 8–10 MHPs. The goal of the focus group interviews is to present, test and discuss preliminary findings and conclusions from the individual interviews. A focus group interview is appropriate for this situation, since the group interaction provides insight into topics of agreement and favourable and unfavourable topics, and experiences with the programme can be shared [55]. A moderator will lead the group discussion, and an assistant moderator will take notes, observe and keep track of the time [56]. The input from the individual interviews will be used in these focus group interviews, to discuss desirable changes to the E-health programme, in order to further improve its quality and usability. In order to obtain new perspectives on the evaluative data from the individual interviews, one half of the focus group participants will not have participated in a previous individual interview. The other half of the focus group members will be purposively selected from the patients and MHPs who previously participated in the individual interviews, where the selection is based on the diverging perspectives on using the relapse prevention programme. These focus group interviews will be conducted in a mental healthcare facility and will take approximately 90 min.

#### Qualitative data analysis

For each of the qualitative research questions, a separate analysis will be performed:

4) What are barriers and facilitators in the implementation of the programme?

The barriers and facilitators in the implementation of the programme will be assessed through the semi-structured interviews and focus group interviews with the MHPs. These interviews will be audio recorded, and transcribed verbatim. Data will be sorted using the CFIR [54], which indicates five domains of implementation (intervention characteristics, outer setting, inner setting, characteristics of individuals and process). Data collection will alternate with data analysis and new topics will be discussed in the following interviews. Two researchers will independently openly code the first three interviews, compare the codes and draft a coding tree. The coding tree will be complemented through the following interviews. This data will be analysed using thematic analysis [57], which means that the interviews will be coded for themes, these codes will then be sorted and analyses will be performed using software programme MaxQDA. When reporting the data, quotations will be used to illustrate the findings.

During this process, multiple researchers will be involved in order to increase the validity and reliability [56]. From the beginning of data collection until the end of data analysis, detailed field notes will be documented, containing specific situations, reflections, and ideas and thoughts of the researchers [56]. In addition, a summary will be made of each interview, containing the most important findings.

5) How do patients evaluate the program?

The evaluation of the programme will be assessed by the semi-structured interviews and focus group interviews with the patients. In addition, in the last follow-up questionnaire (T9), patients will be asked about their satisfaction with the programme and to rate every module they completed on a scale from 0 to 10. The data from the interviews and focus group interviews will be analysed in the same way as described above (research question 4). Data from T9 will be analysed using descriptive statistics.

## Discussion

Providing relapse prevention for anxiety and depression is important, since these disorders are often chronic and recurrent. In this study we will implement and evaluate a newly developed relapse prevention programme, specifically targeted at patients that are (partially) remitted from an anxiety disorder or depression. The mixed-methods approach in this study will provide valuable insights into how patients use the programme, what influences the use of the programme, how usage intensity influences symptoms and how patients evaluate the programme. In addition, information on implementation will be provided which may be relevant for broad implementation of the programme. The findings of this study can be used to further refine and adapt the programme as preferred by patients and MHPs.

A strength of this study is that the relapse prevention programme has been developed based on patient preferences and in close collaboration with patients. Additionally, this programme is not only web-based, but also supplemented by contact with MHPs. Earlier research suggests that E-health can be especially effective when accompanied by therapist contact [58]. By using log data to establish the usage of the programme, the actual use and usage behaviour can be determined [59]. Furthermore, by using mixed methods, a complete view on the use of the programme and its evaluation can be obtained [59].

This design is not appropriate to examine efficacy, since only within-group effect sizes can be determined, which might be considered a limitation of this study. However, the aim of this study was not to examine the efficacy of the programme, but its acceptability. If the programme is acceptable, a randomized controlled trial should be conducted to determine the efficacy of the programme.

## Additional files

Additional file 1: Topic guide interview patient. (DOCX 18 kb)

Additional file 2: Topic guide interview MHP. (DOCX 20 kb)

## Abbreviations

ASI: Anxiety Sensitivity Index
BAI: Beck Anxiety Inventory
CBT: Cognitive behavioural therapy
CFIR: Consolidated framework for implementation research
DSM: Diagnostic and statistical manual of mental disorders
GET READY: Guided E-healTh for RElapse prevention in Anxiety and Depression
IDS-SR: Inventory of Depressive Symptomatology – self-report
MHP: Mental health professional
TiC-P: Trimbos/iMTA Questionnaire for Costs Associated with Psychiatric Illness
WHODAS: World Health Organization Disability Assessment Schedule
