# Supplementary material for: The GET READY relapse prevention programme for anxiety and depression: a mixed-methods study protocol
Source: BMC Psychiatry. 2019 Feb 11;19:64. doi: 10.1186/s12888-019-2034-6 (PMC6371559; doi:10.1186/s12888-019-2034-6)
Supplement: Supplementary file 1 — Topic guide interview patient. (DOCX 18 kb) [file 12888_2019_2034_MOESM1_ESM.docx]

# Additional file 1:Topic guide interview patient

| **Main questions** | **Additional questions** |
| --- | --- |
| Introduction | Sign informed consent  Introduce yourself  Mention the duration of the interview (45 minutes)  Ask permission to audio record the interview, and start the recording  Mention that the name and personal data is saved separately from research data  Mention the purpose of the interview |
| What is your experience with the relapse prevention programme in general? | What were your expectations of the relapse prevention programme?  Could you tell more about the role of the MHP?   - What did you expect from the MHP at the beginning of the study? - How did you experience the contacts? - How did you experience the support from the MHP? - In which way did the MHP help you to stay healthy? - What could be improved in the guidance from the MHP? - Who initiated the contact and how did you experience this?   What is your experience with the E-health programme?   - Usability - Meeting your needs - Pleasure/satisfaction - Available choices - Use of language - Design - Time investment   Did the programme help you to stay healthy/without symptoms?   - In which way did the programme help you to stay healthy?   How do you feel about the combination of E-health and having contact with the MHP? |
| What were the most useful and the least useful parts of the E-health programme? | - Which modules did you complete? What made you choose these modules? - What were your motives concerning using or not using the modules? - Which modules were set up for you, but not completed? - Did you complete the diary? - How much did you use the message function within the programme? - According to the questionnaires, your symptoms have decreased/increased. How did you experience this? |
| What do you like about the relapse prevention programme and what could be improved? | What is the most useful aspect of the relapse prevention programme?  What could be improved in the relapse prevention programme?  What did you miss in the relapse prevention programme? |
| Completion | Are there other topics you would like to discuss?  Do you have any questions?  Would you like to receive the outcomes of the study?  Would you be interested in participating in a focus group interview? |
