# Supplementary material for: The GET READY relapse prevention programme for anxiety and depression: a mixed-methods study protocol
Source: BMC Psychiatry. 2019 Feb 11;19:64. doi: 10.1186/s12888-019-2034-6 (PMC6371559; doi:10.1186/s12888-019-2034-6)
Supplement: Supplementary file 2 — Topic guide interview MHP. (DOCX 20 kb) [file 12888_2019_2034_MOESM2_ESM.docx]

# Additional file 2: Topic guide interview MHP

| **Main questions** | **Additional questions** |
| --- | --- |
| Introduction | Sign informed consent  Introduce yourself  Explain which patient the interview is about  Mention the duration of the interview (45 minutes)  Ask permission to audio record the interview, and start the recording  Mention that the name and personal data is saved separately from research data  Mention the purpose of the interview |
| Practical questions | How many hours a week do you work as MHP?  Do you work in multiple general practices?  How many minutes does a regular contact last?  How many MHPs/GPs are working in this general practice? |
| What is your experience with the relapse prevention programme? | How did you offer the relapse prevention programme to the patient?   - How were follow-up contacts planned? - Who initiated the contact and how did you experience this? - How many contacts did you have? - What did you discuss during the contacts? - Did you stimulate the patient to use the relapse prevention programme? In which way? - How did the programme influence the health of the patient? - To what extent did the programme meet the patients’ symptoms?   What did you expect from the patient at the beginning of the study?  How did you offer the relapse prevention programme to other patients?  *What was it like to offer the relapse prevention programme to the patient?  What is your experience with the E-health programme?   - Usability/structure - Design - Use of language - Time investment - Aspects: what did you use/not use, experience with aspects, relapse prevention plan - Message function/providing feedback |
| What made it easier or harder to implement the relapse prevention programme in the general practice? | The relapse prevention programme itself: intervention characteristics   - What did you think about the quality of the relapse prevention programme? - Did you ever apply relapse prevention strategies before? How does this programme compare to other strategies that you are familiar with? - *How complicated is the intervention?   Factors within the general practice: inner setting   - How could you apply the relapse prevention programme in your ‘normal work’? - Did you experience support from the general practice or GP? How? - In the general practice, what is the willingness to change? - How did having/not having time affect implementation?   Characteristics of individuals   - Did you feel confident offering the relapse prevention programme? Why/why not?   Process of implementation   - Did you experience support from the research team? How?   Influence from outer setting   - Did you have contact with specialised mental healthcare services? What was the effect? |
| What do you like about the relapse prevention programme and what could be improved? | What is the most useful aspect of the relapse prevention programme?  What could be improved in the relapse prevention programme?  *What did you miss in the relapse prevention programme?  Would you use the relapse prevention programme if available after completion of the study? What is needed? |
| Completion | Are there other topics you would like to discuss?  Do you have any questions?  Would you like to receive the outcomes of the study?  Would you be interested in participating in a focus group interview? |

* If not discussed yet, also ask this question
